# A Majority of Human Melanoma Cell Lines Exhibits an S Phase-Specific Defect in Excision of UV-Induced DNA Photoproducts

**DOI:** 10.1371/journal.pone.0085294

**Published:** 2014-01-08

**Authors:** François Bélanger, Vincent Rajotte, Elliot A. Drobetsky

**Affiliations:** Département de Médecine, Université de Montréal and Centre de Recherche, Hôpital Maisonneuve Rosemont, Montréal, Québec, Canada; University of Queensland Diamantina Institute, Australia

## Abstract

It is well established that efficient removal of highly-promutagenic UV-induced dipyrimidine photoproducts *via* nucleotide excision repair (NER) is required for protection against sunlight-associated malignant melanoma. Nonetheless, the extent to which reduced NER capacity might contribute to individual melanoma susceptibility in the general population remains unclear. Here we show that among a panel of 14 human melanoma strains, 11 exhibit significant inhibition of DNA photoproduct removal during S phase relative to G0/G1 or G2/M. Evidence is presented that this cell cycle-specific NER defect correlates with enhanced apoptosis and reduced clonogenic survival following UV irradiation. In addition, melanoma strains deficient in S phase-specific DNA photoproduct removal manifest significantly lower levels of phosphorylated histone H2AX at 1 h post-UV, suggesting diminished activation of ataxia telangiectasia and Rad 3-related (ATR) kinase, i.e., a primary orchestrator of the cellular response to UV-induced DNA replication stress. Consistently, in the case of DNA photoproduct excision-proficient melanoma cells, siRNA-mediated depletion of ATR (but not of its immediate downstream effector kinase Chk1) engenders deficient NER specifically during S. On the other hand simultaneous siRNA-mediated depletion of ataxia telangiectasia mutated kinase (ATM) and DNA-dependent protein kinase catalytic subunit (DNA-PKcs) exerts no significant effect on either phosphorylation of H2AX at 1 h post-UV or the efficiency of DNA photoproduct removal. Our data suggest that defective NER exclusively during S phase, possibly associated with decreased ATR signaling, may constitute an heretofore unrecognized determinant in melanoma pathogenesis.

## Introduction

During the past five decades the incidence of malignant melanoma has been rising steadily at an alarming rate in Caucasian populations worldwide [1]. Lifetime risk in the US is currently 1/50, with 76,690 new cases expected in 2013 (Cancer Facts and Figures 2013, http://www.cancer.org/cancer/skincancer-melanoma/detailedguide/melanoma-skin-cancer-key-statistics, accessed February 28, 2013). Moreover melanoma can strike in the prime of life and is often associated with dismal prognosis. Indeed primary melanomas, unless diagnosed early and promptly resected, tend to metastasize aggressively at which point the disease is generally refractory to therapeutic intervention [2].

It is now well established that exposure to UV originating from either natural sunlight or tanning beds represents a preeminent environmental risk factor for melanoma [3], [4]. This is attributable to UV induction in target melanocytes of highly-genotoxic dipyrimidine DNA photoproducts, i.e., cyclobutane pyrimidine dimers (CPDs) and 6–4 pyrimidine-pyrimidone photoproducts (6–4PPs), that distort the DNA helix and strongly block DNA replication and transcription [5]. In humans nucleotide excision repair (NER) is the only pathway for removing helix-distorting adducts including CPDs and 6–4PPs, and thereby constitutes an essential frontline defense against melanomagenesis. This is underscored by the rare autosomal recessive disease *Xeroderma pigmentosum* (XP) characterized by inactivating mutations in various NER pathway genes (*XP-A* through *-G*) and up to 10,000-fold increased risk of developing skin cancers including melanoma [6].

The exquisite sensitivity of XP patients to cutaneous tumourigenesis raises the distinct possibility that germline polymorphisms in NER genes which confer reduced capacity to eliminate UV-induced DNA adducts (though not sufficiently to cause XP) might underlie higher individual susceptibility to melanoma in the general population. Moreover alterations in NER genes that severely compromise repair might be expected to arise stochastically at relatively high frequency in individual melanocytes during lifetime exposure to promutagenic UV, thereby driving sporadic melanomagenesis. Although epidemiological associations between *XP-C*, *-D*, and *-F* polymorphisms and increased melanoma risk have been reported [7], [8], [9], [10], no firm evidence to our knowledge supports a major role for NER pathway gene mutations in either familial or sporadic melanoma. Concerning regulatory mechanisms upstream of NER, it has been clearly shown that a functional p53 tumour suppressor pathway, triggered following genotoxic insult to regulate apoptosis and growth arrest, is also required for efficient CPD removal in various cell types including melanoma [11], [12]; nonetheless mutational inactivation of *p53* appears rare in melanoma [13]. On the other hand the PTEN tumour suppressor, frequently downregulated by genetic or epigenetic means in melanoma [14], has recently been implicated in protection against UVB-induced nonmelanoma skin cancer by positively regulating NER [15]. However the extent to which PTEN might similarly influence UV damage repair in the context of malignant melanoma was not evaluated.

DNA replication stress including that generated by UV-induced DNA photoproducts is accompanied by early activation of the ataxia telangiectasia and Rad 3-related (ATR) kinase which rapidly phosphorylates several hundred protein substrates [16]. An important fraction of these ATR targets then cooperates to promote DNA synthesis restart through resolution of stalled replication forks at sites of UV damage, that in turn guards against replication fork collapse and secondary formation of highly-lethal DNA double-strand breaks [17]. Consistent with such a role for ATR in the maintenance of genomic stability during DNA replication, our laboratory previously provided evidence that ATR status is a critical determinant of NER efficiency exclusively during S phase of the cell cycle. Indeed we showed in primary lung fibroblasts depleted for ATR, or in *ATR*-deficient Seckel syndrome skin fibroblasts, that excision of UV photoproducts is significantly inhibited in a p53-independent manner during S whereas photoproduct removal during G0/G1 and G2/M appears normal [18]. (Hereafter excision of UV photoproducts via NER occurring specifically in S phase will be denoted SPR; S-Phase Repair). Interestingly we also showed that 50% among a group of randomly-chosen human tumour cell lines manifest an S phase-specific reduction in NER capacity, permitting speculation that such a defect might constitute a relatively common feature of human cancers. Towards addressing this latter possibility, we now demonstrate that among a collection of 14 human melanoma cell lines, 11 exhibit significant impairment in removal of UV-induced DNA photoproducts during S phase relative to G0/G1 or G2/M. Moreover this SPR deficiency is shown to correlate with reduced levels of phosphorylated histone H2AX post-UV, suggestive of diminished ATR kinase activation. Our data indicate the presence of a characteristic S phase-specific NER defect in human melanoma cell lines, and thus might harbour important implications for melanoma development.

## Materials and Methods

### Cell culture

Fourteen human melanoma strains from the Wistar collection (WM35, WM1366, WM1341D, WM3248, WM902B, WM3211, WM1158, WM278, WM793B, WM1361A, WM1617, WM3734, WM2664, WM983B), and human primary XPA-deficient skin fibroblasts (GM01630), were purchased from the Coriell Institute (Camden, NJ). The hTERT-immortalized skin fibroblast lines F02-98 and 1BR, derived from a Seckel syndrome patient and normal individual, respectively, were kindly provided by Dr. P. Jeggo (University of Sussex) [19]. All culture media and supplements used in this study were purchased from Invitrogen (Burlington, Canada). The above strains were propagated in Eagle's MEM containing 15% FBS, essential and nonessential amino acids, vitamins, L-glutamine, and antibiotics. Human primary melanocytes (strains GM22134, GM21807, and GM22141; Coriell Institute) were grown in Medium 254 containing human melanocyte growth supplement.

### Cell irradiations

Cell monolayers were washed with PBS and covered with 1 ml of PBS, followed by irradiation with either monochromatic 254-nm UVC using a G25T8 germicidal lamp (Philips), or polychromatic UVB (290-320-nm) using an F15T8 lamp (UVP). The incident UVB was passed through a 2-mm thick Schott WG305 filter to vastly attenuate wavelengths below 290-nm, as previously described [20]. The fluences were 0.1 J/m^2^/s for 254-nm UVC, and 2.5 J/m^2^/s for UVB, as measured with a Spectroline DRC 100× digital radiometer equipped with DIX-254 and DIX-300 sensors, respectively. In the case of ionizing radiation, cells were treated with a ^137^Cs source (Gamma Cell; Atomic Energy Canada) at a dose rate of 6.3×10^−2^ Gy/s.

### Evaluation of cell cycle progression

Exponentially-growing cultures were irradiated with 15 J/m^2^ of 254-nm UVC, followed immediately by incubation with 30 µM BrdU (Sigma-Aldrich, Oakville, Canada) for 1 h. Mock-irradiated cells were BrdU-labeled for 30 min. Monolayers were washed thoroughly with PBS, and fresh complete medium added. Cells were harvested at indicated times, fixed in ethanol, and denatured with 2N HCl plus 0.5% Triton X-100. Cells were then stained with AlexaFluor647-conjugated anti-BrdU antibody (Invitrogen, Burlington, Canada) and 5 μg/ml PI (Invitrogen, Burlington, Canada). Samples were analyzed using a FACSCalibur flow cytometer in conjunction with FlowJo software (Becton-Dickinson, Mississauga, Canada).

### Determination of NER efficiency as a function of cell cycle

The excision of 6–4PPs was quantified in each of G0/G1, S, and G2/M as described previously [18]. Briefly replicate exponentially-growing cultures (1×10^6^ cells on 60-mm dishes) were mock-irradiated or irradiated with 15 J/m^2^ of 254-nm UVC or 300 J/m^2^ UVB, and harvested either immediately or following 6 h incubation at 37°C in normal medium. Cells were then fixed, denatured with 0.2N HCl plus 0.5% Triton X-100, and stained with FITC-conjugated anti-6-4PP antibody (Kamiya, Seattle, WA) and PI. Flow cytometry was employed to analyze the extent of 6–4PP removal at 6 h post-UV for populations gated in each phase of the cell cycle.

Removal of CPDs as a function of cell cycle was also evaluated using an adaptation of the above protocol based on triple labeling as described previously [21]. Briefly exponentially-growing cultures were irradiated with either 10 J/m^2^ of 254-nm UVC or 200 J/m^2^ UVB, followed immediately by incubation with 30 µM BrdU (Sigma-Aldrich, Oakville, Canada) for 1 h. Monolayers were then washed thoroughly with PBS and fresh complete medium added. For the 0 h time-point, BrdU was added 30 min prior to UV irradiation, after which cells were immediately harvested for analysis. Following further incubation for either 12 or 24 h cultures were harvested, fixed in ethanol, and denatured with 2N HCl plus 0.5% Triton X-100. Cells were then stained with anti-CPD antibody (Kamiya, Seattle, WA), AlexaFluor647-conjugated anti-BrdU antibody and PI, and analyzed by flow cytometry.

### Determination of clonogenic survival and sub-G1 DNA content in SPR-proficient vs -deficient melanoma cell lines post-UV

Exponentially-growing cultures on 60-mm dishes were irradiated with 5, 10, or 15 J/m^2^ of 254-nm UVC, or mock-irradiated, and immediately trypsinized for plating of appropriate cell numbers on 100-mm dishes in fresh medium. Following 21 days incubation, colonies were stained with 0.5% methylene blue (w/v) in 50% methanol (v/v). Survival is expressed as a percentage relative to mock-irradiated cells.

For quantification of sub-G1 peak, exponentially-growing cultures on 60-mm dishes were irradiated with 10 J/m^2^ UVC and harvested at 24, 48, and 72 h post-UV. Mock-irradiated cells were used as the 0 h time point. Cells were fixed in ethanol, stained with PI, and analyzed by flow cytometry.

### Quantification of γH2AX expression

γH2AX was quantified in intact cells by flow cytometry using a method adapted from Marti *et al*. [22]. Exponentially-growing cultures (1×10^6^ cells on 60-mm dishes) were mock-irradiated or irradiated with 15 J/m^2^ of 254-nm UVC and immediately refed with normal culture medium. At 1 h post-UV cells were trypsinized and briefly resuspended in 3 ml of PBS containing soybean trypsin inhibitor (Invitrogen, Burlington, Canada), washed with PBS, and resuspended for 15 min in 3 ml ice-cold PBS plus 1% formaldehyde. Cells were then washed with PBS, fixed in 3 ml 75% ice-cold ethanol, and incubated at −20°C for 2 h. Fixed cells were washed with PBS-TB (PBS plus 0.2% triton X-100/1% BSA) and resuspended in 0.2 ml PBS-TB containing 1∶200 primary anti-γH2AX (Ser 139) antibody. Following overnight incubation at 4°C cells were washed with PBS-TB and resuspended in 0.2 ml PBS-TB containing 1∶100 FITC-conjugated rabbit anti-mouse antibody (Sigma-Aldrich, Oakville, Canada) for 1 h at room temperature. Cells were washed with PBS-TB, resuspended in 0.35 ml PBS containing 5 μg/ml PI plus 100 μg/ml RNase A, and incubated for 30 min at room temperature. Analysis was carried out by flow cytometry.

### Western Blotting

Western blotting was performed as previously described [23]. Commercially-obtained antibodies against the following proteins were used: (i) ATR (sc-1887), ATM (sc-23921), Chk1 (sc-56291), p-Chk1/Ser345 (sc-17922R), DNA-PKcs (sc-1552), and YY1 (sc-1703) from Santa Cruz (Santa Cruz, CA); (ii) ATRIP (2737) from Cell Signaling Technology (Danvers, MA); (iii) actin (ab8227-50) from Abcam (Toronto, Canada); (iv) GAPDH (MAB374) and γH2AX/Ser139 (JBW301) from Millipore (Billerica, MA).

### siRNA-mediated depletion of ATR, Chk1, ATM, and DNA-PKcs

siRNA smartpools targeting ATR, Chk1, ATM, DNA-PKcs, or non-targeting control siRNA (Dharmacon, Lafayette, CO.) were transfected into cells at 50–70% confluence using Lipofectamine 2000 according to the manufacturer's protocol (Invitrogen, Burlington, Canada). Cells were re-transfected 1 day later and further incubated for 48 h prior to use.

## Results

### A majority of UV-irradiated human melanoma cell lines manifests defective SPR

**Figure 1:**
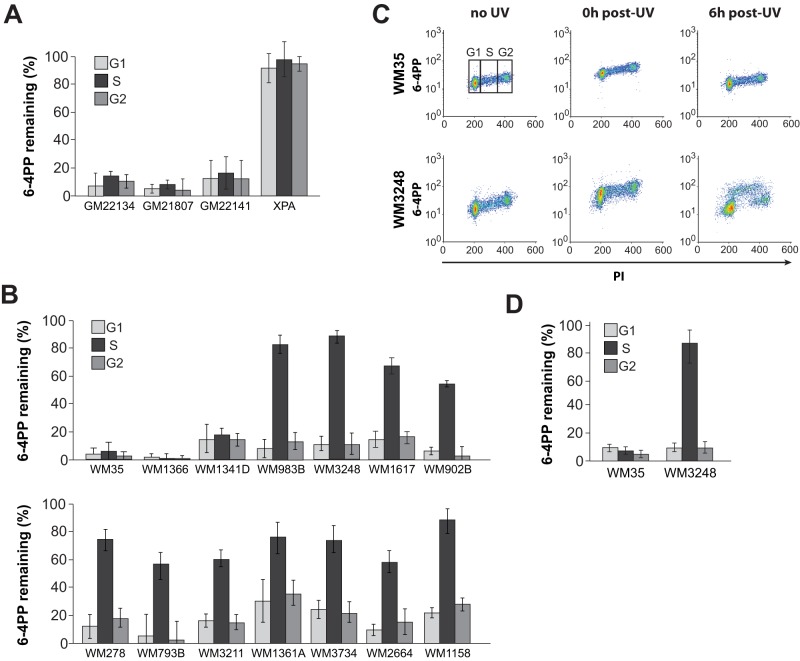
Cell cycle-specific 6–4PP excision in normal melanocytes and melanoma cell lines. **A**) Graphical representation of 6–4PP removal as a function of cell cycle at 6 h post-UVC for 3 primary melanocyte strains and XPA-deficient skin fibroblasts. **B**) Same as A, but for 14 melanoma strains. In the case of all SPR-deficient strains, excision during S phase is significantly slower relative to other phases (p<0.01; two-tailed paired t-test). **C**) Representative bivariate dot plots showing WM35 and WM3248 (SPR-proficient and -deficient, respectively) either UV- or mock-irradiated as indicated, and stained with PI and anti-6-4PP antibody. Cells were gated in each phase of the cell cycle as shown for WM35 (no UV). **D**) Graphical representation of 6–4PP excision as a function of cell cycle at 6 h post-UVB (290–320-nm; 300 J/m^2^) in WM35 and WM3248. For all panels in this figure, values represent the mean ± SEM of three independent experiments.

Fourteen human melanoma strains (Table S1) and three primary melanocyte lines were exposed to 15 J/m^2^ of 254-nm UVC and the extent of 6–4PP removal at 6 h post-UV was quantified in each of G0/G1, S, and G2/M using a flow cytometry-based NER immunoassay previously developed in our laboratory [18]. (Unless otherwise indicated all irradiations in this study were performed with 15 J/m^2^ of 254-nm UVC; hereafter UVC). All primary melanocyte lines exhibited >80% 6–4PP removal by 6 h post-UV during all phases (Fig. 1A) consistent with previous studies in human skin fibroblasts demonstrating similarly rapid repair of this photoproduct [24]. Control XPA-deficient primary skin fibroblasts were, as expected, defective in NER of 6–4PPs during the entire growth cycle (Fig. 1A). Remarkably, while three of the melanoma strains manifested characteristically rapid excision of 6–4PPs, the remaining 11 were significantly deficient in removal of this photoproduct in S relative to either G0/G1 or G2/M (Fig. 1B). Representative bivariate dot plots showing raw data on 6–4PP excision for two melanoma strains, i.e., SPR-proficient WM35 and SPR-deficient WM3248, are presented in Figure 1C.

We emphasize that 15 J/m^2^ of the widely-used model mutagen 254-nm UVC induces a yield of dipyrimidine photoproducts equivalent to that produced during 1–2 h exposure to natural sunlight [25], [26]; however UVC is strongly absorbed by stratospheric ozone and thus vastly attenuated at the earth's surface. In addition studies have indicated that cellular responses to UV including mutagenesis and DNA repair can vary as a function of wavelength [20], [27]. We therefore irradiated SPR-proficient WM35 and SPR-deficient WM3248 with a filtered polychromatic UVB source emitting 290–320-nm, i.e. closely approximating the primary genotoxic/carcinogenic portion of the terrestrial solar spectrum. The dose of UVB employed (300 J/m^2^) induced a yield of 6–4PPs similar to that generated by 15 J/m^2^ of UVC; moreover the cell cycle-specific nucleotide excision capacities of WM35 or WM3248 were very similar in response to either UVC or UVB (compare Figs. 1B and 1D).

**Figure 2:**
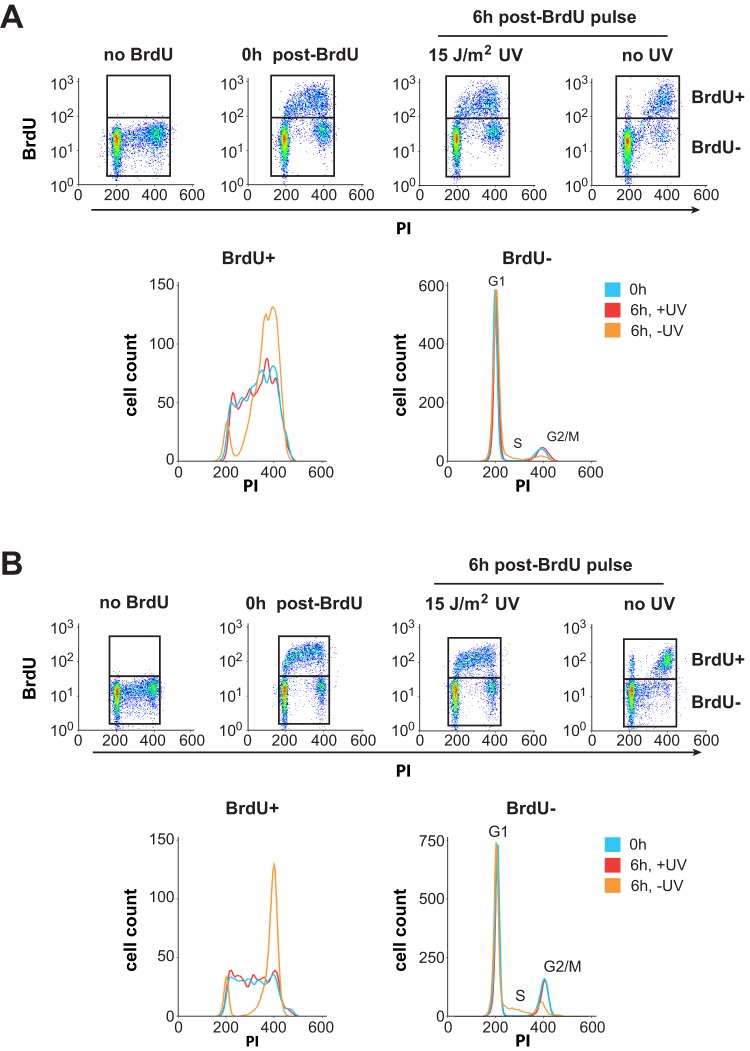
Cell cycle progression in melanoma cells post-UVC. Cells were irradiated with UVC or mock-irradiated and then pulse-labeled with BrdU to mark cells in S-phase at the time of irradiation. Cultures were harvested immediately (0 h) or further incubated for 6 h. Cells were stained with PI and anti-BrdU (Alexa-647), and analyzed by flow cytometry. **A**) SPR-proficient WM35. BrdU+ and BrdU- populations were gated as shown in the bivariate dot plots (top panels). Histograms represent DNA content of BrdU+ and BrdU- cells under each condition (lower panels). **B**) Same as A, but for SPR-deficient WM3248. Similar data for all other melanoma cell lines used in this study are presented in Figure S1.

We note that under our experimental conditions cell cycle progression appears to be arrested in all melanoma strains for at least 6 h post-UVC. SPR-proficient WM35 (Fig. 2A) and SPR-deficient WM3248 (Fig. 2B) were pulse labeled with BrdU to distinguish cells in S-phase (BrdU+) from ones in G1 or G2/M (BrdU−) at the time of irradiation. In the absence of UVC BrdU+ cells progressed rapidly into late S and G2/M; moreover a small fraction of BrdU− cells appeared to move from G1 into S and from G2/M into G1 (as judged by the reappearance of cells in early S and decrease in G2/M cells). In contrast, at 6 h post-UVC neither WM35 nor WM3248 displayed obvious progress in any phase of the cell cycle. Figure S1 depicts similar cell cycle profiles for all other melanoma strains used in this study. These data indicate that variations between melanoma strains in the recovery of cell cycle progression post-UVC did not interfere with accurate comparative quantification of 6–4PP removal during different phases.

**Figure 3:**
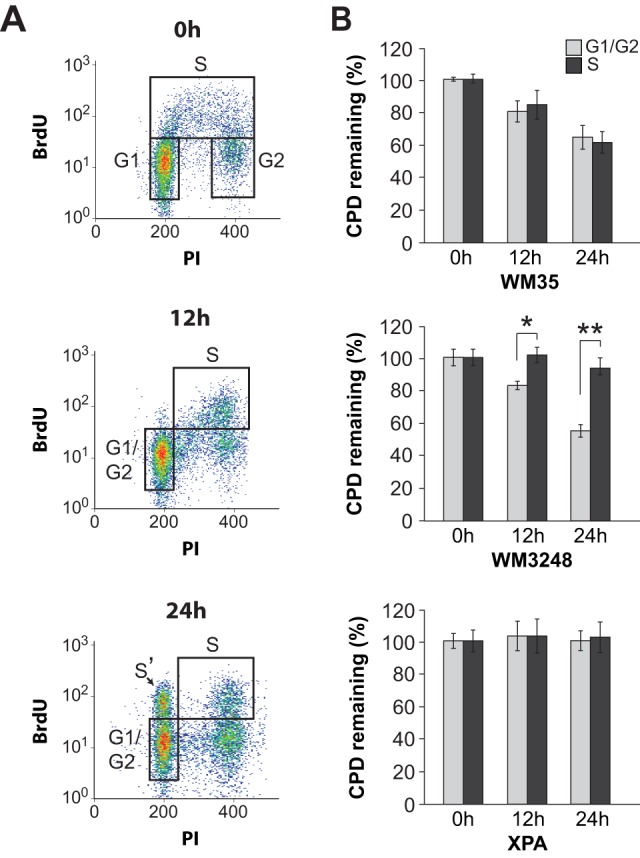
Cell cycle-specific CPD excision in melanoma cell lines. Cells were irradiated with 10/m^2^ UVC and then labeled with BrdU for 1 h (except for the 0 h time point, where BrdU labeling was performed for 30 min prior to UV). At the indicated times post-UV, cells were stained with anti-CPD (FITC), PI, and anti-BrdU (Alexa-647) and analyzed by flow cytometry. **A**) Bivariate plots of WM35 showing the distribution of BrdU-positive cells at different times post-UV. The S' population indicated with an arrow at 24 h post-UV have traversed mitosis after increasing their DNA content and are excluded from the analysis. The extent of CPD removal is compared for cells in S phase at the time of irradiation (designated S), vs. cells in G1 at the time of irradiation which includes a minor proportion of cells that were initially in G2 and have migrated into the G1 compartment during the post-UV incubation period (designated G1/G2) (see text for details). **B**) Graphical representation of CPD excision in WM35 (top), WM3248 (middle), and XPA control skin fibroblasts (bottom). * and **, two-tailed paired t-test comparing CPD excision during S-phase vs G1/G2; p<0.02 and 0.001 at 12 and 24 h, respectively. For all panels in this figure, values represent the mean ± SEM of three independent experiments.

We also evaluated removal of CPDs as a function of cell cycle in human melanoma strains. These photoproducts, which distort the DNA helix considerably less than 6–4PPs, are not recognized as efficiently by the NER machinery and hence repaired much more slowly (generally 40–50% removal at 24 h post-UV). This renders cell cycle-specific quantification of CPD removal by our method more challenging, i.e., compared to the situation for 6–4PPs where excision is monitored over a 6 h post-UVC incubation period during which DNA synthesis and cell growth are strongly inhibited by the UVC treatment. Thus in the case of CPDs it is essential to control for cell cycle progression during considerably longer post-UVC incubations up to 24 h. For this purpose we employed a triple labeling protocol utilizing BrdU, i.e., in addition to PI and anti-CPD antibody, to mark and follow cells that were in S phase at the time of irradiation [21]. Representative bivariate plots (Fig. 3A) show the distribution of BrdU+ cells gated at various times after treatment with 10 J/m^2^ UVC for strain WM35. The S' population appearing at 24 h (indicated with an arrow; Fig. 3A, lower panel) represents BrdU+ cells that have traversed mitosis after increasing their DNA content, therefore requiring exclusion from the analysis. We also emphasize that under our experimental conditions some cycling of cells restarts by approximately 12 h post-UV; thus it is not possible to unequivocally distinguish the G0/G1 and G2/M populations at 12 and 24 h. Indeed the populations designated “G1/G2” at these time points contain some cells that were in G2 at the time of irradiation. However these cells did not increase their DNA content before dividing, and as such do not interfere with our analysis. Consistent with the results on 6–4PP shown in Figure 1B, for WM35 no difference in the extent of CPD removal was noted between the S and G1/G2 populations (Fig. 3B, upper panel), whereas WM3248 exhibited significant inhibition of CPD excision during S phase relative to G1/G2 (Fig. 3B, middle panel). Control XPA-deficient skin fibroblasts, as expected, were defective in CPD removal during all phases at 12 and 24 h post-UVC (Fig. 3B, lower panel).

### Reduced clonogenic survival and increased sub-G1 content in SPR-deficient vs -proficient melanoma cell lines following UV irradiation

**Figure 4:**
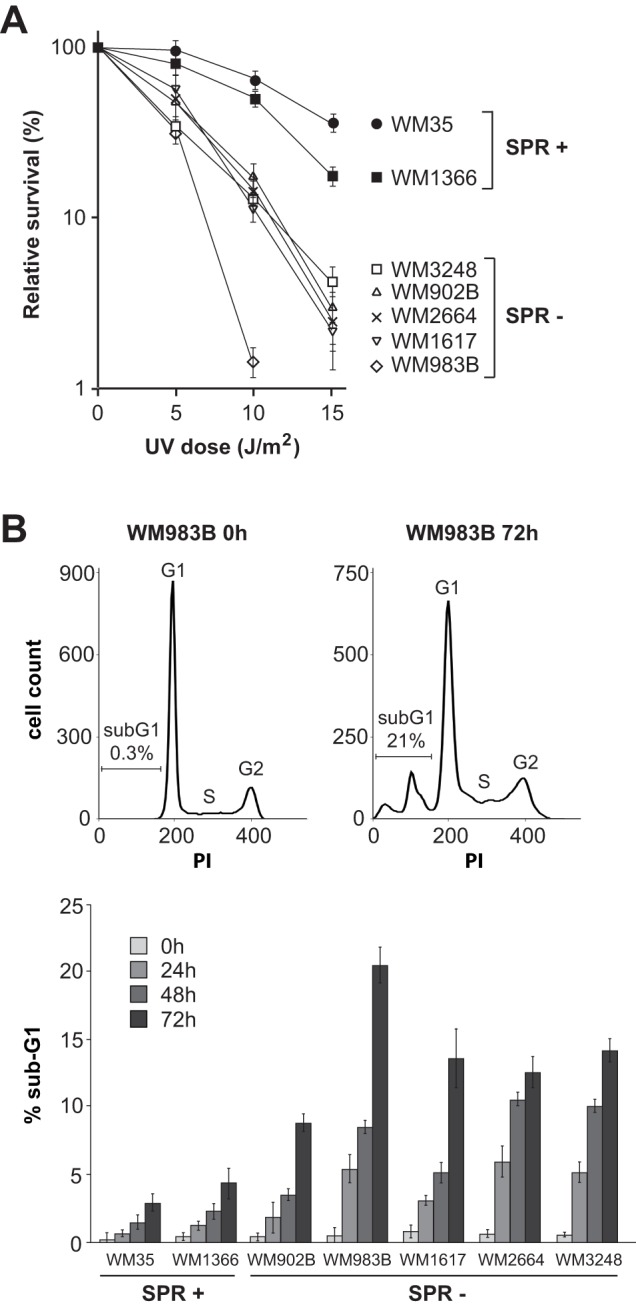
Determination of clonogenic survival and sub-G1 DNA content in SPR+ vs SPR- strains post-UVC. **A**) Clonogenic survival in response to various doses of UVC was calculated as the number of surviving colonies (containing at least 50 cells) for irradiated cells relative to mock-irradiated counterparts. Values represent the mean +/− SEM of four independent experiments. **B**) Percentage of sub-G1 cells at different times after exposure to 10 J/m^2^ of UVC in melanoma cell lines. Representative cell cycle profiles are shown for the SPR-deficient strain WM983B at 0 h and 72 h (top). Values represent the mean +/− SEM of three independent experiments.

Clonogenic survival was determined as a function of UV dose in two SPR-proficient versus five SPR-deficient melanoma strains. In accord with the well-established role of NER in mitigating UV-induced cell death, SPR-proficient WM35 and WM1366 were significantly less sensitive to the cytotoxic effects of UV compared to all SPR-deficient counterparts tested (Fig. 4A). We note that all the other melanoma strains among our collection exhibit very low cloning efficiencies in the absence of genotoxic stress, and therefore could not be evaluated by colony-forming assay.

Increased cell death following UV irradiation is generally associated with enhanced apoptosis. Quantification of sub-G1 DNA content was used to evaluate the possibility that SPR-deficient melanoma strains are more sensitive to UV-induced apoptosis compared with SPR-proficient counterparts. The same melanoma cell lines evaluated above for clonogenic survival were treated with 10 J/m^2^ UVC and the proportion of sub-G1 cells determined at 0 (mock-irradiated), 24, 48, and 72 h (Fig. 4B). For each strain this proportion increased with time; however SPR-deficient strains manifested higher proportions of sub-G1 cells compared to SPR-proficient counterparts, consistent with increased rates of UV damage-induced apoptosis.

### H2AX phosphorylation is significantly reduced in SPR-deficient vs. -proficient melanoma strains at 1 h post-UV

**Figure 5:**
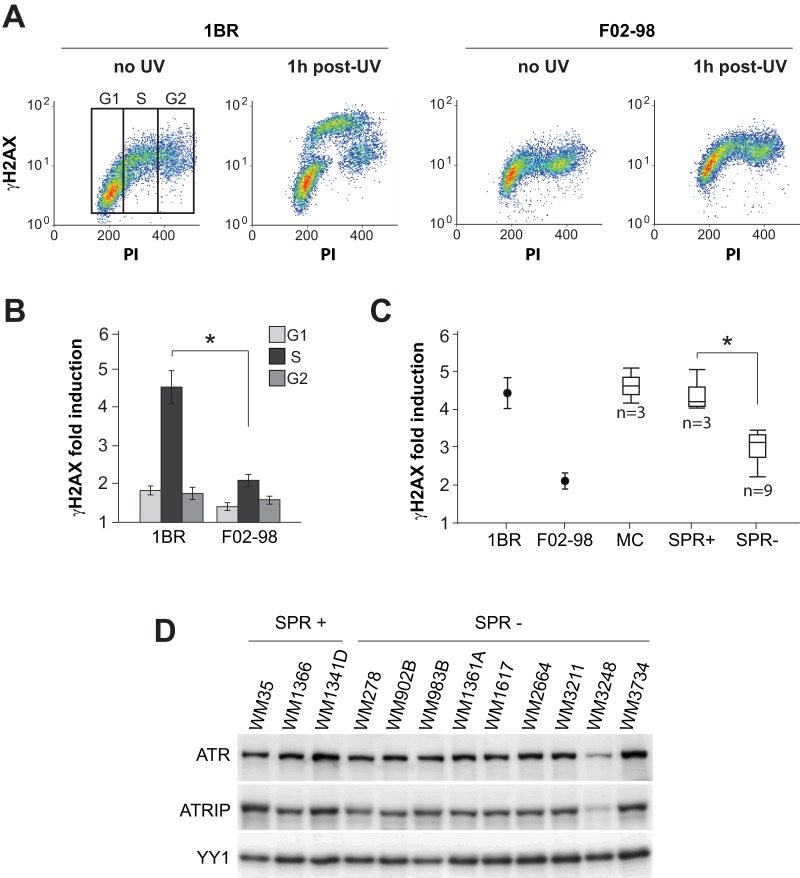
Correlation between defective SPR and reduced H2AX phosphorylation in melanoma cell lines. **A**) Representative bivariate dot plots illustrating relative γH2AX induction as a function of cell cycle at 1 h post-UV in ATR-proficient 1BR vs. the ATR-hypomorphic counterpart F02-98. Cells were gated in each phase of the cell cycle as shown for 1BR (no UV). **B**) Graphical representation of γH2AX induction as a function of cell cycle for 1BR and F02-98 at 1 h post UV. * p<0.001; two-tailed unpaired t-test comparing mean γH2AX induction in S-phase for 1BR vs F02-98. **C**) Graphical representation of γH2AX induction during S phase in 1BR vs F02-98 (same as panel B), compared with 3 normal melanocyte lines (MC), 3 SPR+ melanoma strains and 9 SPR- melanoma strains. Values for melanocytes and melanoma strains are illustrated as a box plot. * p<0.002; two-tailed unpaired t-test comparing mean γH2AX induction for SPR+ vs SPR- strains. **D**) ATR and ATRIP protein levels were determined in whole cell extracts for various melanoma strains by western blot. YY1 is used as a loading control.

We next investigated whether defective SPR in melanoma cell lines might be correlated with reduced ATR activity. Following exposure to UV, histone H2AX is rapidly phosphorylated on Ser139 in an ATR-dependent manner primarily during S phase, and this event is recognized as a reliable indicator of ATR kinase activation [22], [28]. In order to quantify the induction of phosphorylated H2AX (γH2AX) as a function of cell cycle in UV-irradiated melanoma strains, we employed a sensitive flow cytometry-based approach. As reference controls, two h-TERT immortalized skin fibroblast lines were included in the analysis: (i) 1BR, obtained from a normal ATR-proficient individual, and (ii) F02-98, derived from a patient afflicted with Seckel syndrome, characterized by hypomorphic ATR deficiency [19]. Representative bivariate plots (Fig. 5A) show raw data for 1BR and F02-98 which were used to calculate fold γH2AX inductions from the mean fluorescence in each phase of the cell cycle. As expected, induction of γH2AX was most prominent during S-phase in both strains, and was significantly lower in ATR-deficient F02-98 relative to ATR-proficient 1BR (Fig. 5B).

Figure 5C illustrates S-phase-specific γH2AX fold-inductions at 1 h post-UV for 1BR and F02-98, compared with 3 normal melanocyte lines (GM22134, GM21807 and GM22141) and 12 melanoma strains, i.e. 3 SPR-proficient (WM1341D, WM35 and WM1366) vs. 9 SPR-deficient (WM3734, WM3211, WM1617, WM983B, WM3248, WM902B, WM1361A, WM278 and WM2664). Absolute values for all strains are listed in Table 1. Fold γH2AX inductions for 1BR and F02-98 were 4.6±0.5 and 2.0±0.2, respectively. Of note, the 3 SPR-proficient melanoma strains exhibited significantly higher fold-inductions (range 4.0±0.3–5.0±0.7) relative to 9 SPR-deficient counterparts (range 2.2±0.1–3.4±0.3) (p<0.002, student t test). Normal melanocytes exhibited γH2AX induction levels comparable to those of SPR-proficient melanoma strains (range 4.2±0.2–5.1±0.5).

**Table 1:** Induction of γH2AX during S-phase as a function of SPR status.

Cell type	Strain	γH2AX	SPR
Fibroblast	1BR	4.6±0.5	+
Fibroblast	F02-98	2.0±0.2	−
Melanocyte	GM22141	5.1±0.5	+
Melanocyte	GM21807	4.6±0.3	+
Melanocyte	GM22134	4.2±0.2	+
Melanoma	WM1366	5.0±0.7	+
Melanoma	WM35	4.1±0.3	+
Melanoma	WM1341D	4.0±0.3	+
Melanoma	WM2664	3.4±0.3	−
Melanoma	WM278	3.3±0.4	−
Melanoma	WM1361A	3.3±0.3	−
Melanoma	WM902B	3.2±0.3	−
Melanoma	WM3248	3.1±0.3	−
Melanoma	WM983B	3.0±0.4	−
Melanoma	WM1617	2.7±0.2	−
Melanoma	WM3211	2.3±0.2	−
Melanoma	WM3734	2.2±0.1	−

Fold induction of γH2AX in 1BR normal skin fibroblasts, F02-98 Seckel syndrome skin fibroblasts, 3 normal human melanocyte cell lines, and 12 human melanoma cell lines was measured at 1 h post-UVC by flow-cytometry as depicted in Figure 5. Values represent the mean +/− SEM of three independent experiments.

The above data together with our previous results in primary human lung fibroblasts cited earlier suggest that modest but significant reductions in histone H2AX phosphorylation during S phase, possibly indicating downregulation of ATR signaling, may underlie the S phase-specific NER defect observed here in a majority of human melanoma cell lines. We compared ATR protein levels in the SPR-proficient vs -deficient melanoma strains by Western blotting (Fig. 5D). No obvious differences were noted, except in the case of SPR-deficient WM3248 which appeared to exhibit markedly reduced ATR levels. Moreover as would be expected (see Discussion), this strain also exhibited very low expression of ATR Interacting Protein (ATRIP).

### Downregulation of ATR but not of ATM or DNA-PK causes reduced H2AX phosphorylation and defective SPR in UV-irradiated melanoma cells

ATR is a member of the PI3 kinase-related kinase family which also includes ATM and DNA-PK. As cited above, it has been shown that ATR rapidly phosphorylates H2AX predominantly during S phase following UV-induced replication stress. On the other hand ATM and DNA-PK are known to induce γH2AX in a similarly rapid manner, but during all cell cycle phases in response to DNA double-strand breaks generated after exposure to clastogens such as ionizing radiation (IR) [29], [30]. Despite these distinctions, it was important to evaluate any potential contribution of ATM or DNA-PK to S phase-specific NER. We initially treated the three SPR-proficient melanoma strains in our collection with (i) 10 mM caffeine known to inhibit both ATR and ATM, or (ii) 30 µM wortmannin which inhibits ATM and DNA-PK but not ATR [31], [32] followed by exposure to IR or UV. At 30 min following IR treatment γH2AX was induced in each SPR-proficient melanoma strain during all phases of the cell cycle, and this was inhibited by both caffeine and wortmannin (Fig. S2A). However at 1 h post-UVC γH2AX was induced primarily during S, and this was abrogated by caffeine whereas wortmannin had no apparent effect (Fig. S2B). Moreover caffeine but not wortmannin significantly inhibited 6–4PP excision exclusively in S-phase (Fig. S2C).

**Figure 6:**
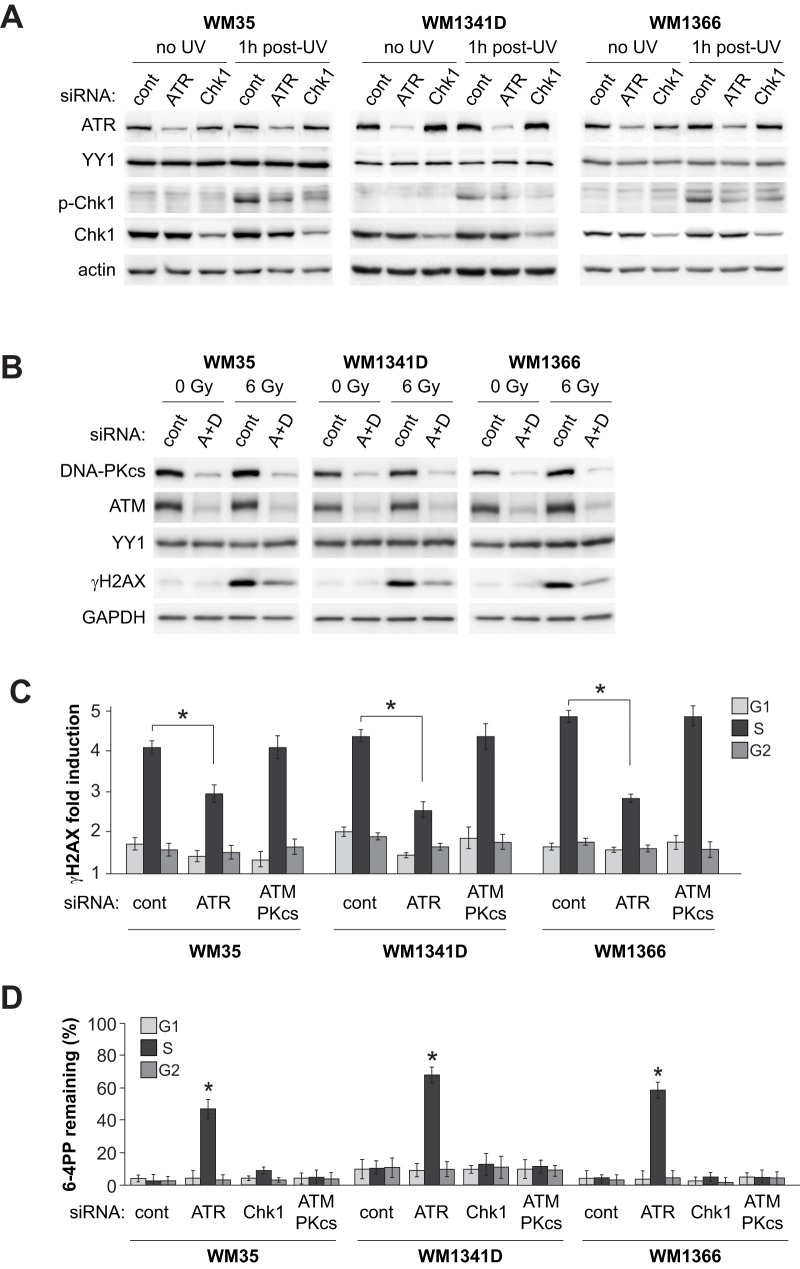
Downregulation of ATR but not Chk1 engenders defective SPR in melanoma cells. **A**) Each of the three SPR-proficient strains in our collection were incubated with siRNA pools against ATR or Chk1, or with non-targeting control siRNA, and treated with UVC or mock-irradiated. Protein levels for ATR, Chk1, and p-Chk1(S345) were determined by western blotting. YY1 and actin were used as loading control for ATR and Chk1, respectively. **B**) SPR-proficient strains were incubated with combined siRNA pools targeting both ATM and DNA-PKcs (A+D) or with non-targeting control siRNA, and treated with 6 Gy of IR or mock-irradiated. Protein levels for ATM, DNA-PKcs, and γH2AX were determined by western blotting. YY1 and GAPDH were used as loading controls for ATM/DNA-PKcs and γH2AX, respectively. **C**) γH2AX induction as a function of cell cycle at 1 h post-UV for siATR knockdown and ATM/DNA-PKcs double knockdown vs. non-targeting siRNA control. * p<0.01, two-tailed unpaired t-test comparing γH2AX induction in S-phase for siATR knockdown cells vs. non-targeting siRNA control. **D**) Cell cycle-specific excision of 6–4PP at 6 h post-UV in ATR, Chk1 and ATM/DNA-PK siRNA knockdown cells vs. non-targeting siRNA control. * p<0.005, two-tailed paired t-test comparing the extent of 6–4PP removal in G1 vs S. For all panels in this figure, values represent the mean ± SEM of three independent experiments.

The above experiments using pharmacological inhibitors may suggest that induction of γH2AX at 1 h post-UVC, as well as S phase-specific NER, are regulated by ATR but not by ATM or DNA-PK. However it is important to emphasize that both caffeine and wortmannin exhibit non-specific effects involving other kinases. We therefore treated our three SPR-proficient melanoma strains with siRNA pools specifically targeting DNA-PKcs (DNA-PK catalytic subunit), ATM, ATR, or Chk1. The latter is a direct target and critical effector kinase of the ATR pathway during the cellular response to UV-induced replication stress [17]. For all three SPR-proficient strains, knockdown of either ATR or Chk1 resulted in diminished Chk1 phosphorylation at 1 h post-UVC (Fig. 6A). Simultaneous depletion of both ATM and DNA-PKcs caused a marked decrease of H2AX phosphorylation 30 min after treatment with 6 Gy of IR, as evaluated by western blotting (Fig. 6B). Moreover consistent with our data presented thus far, knock-down of ATR but not of ATM and DNA-PKcs significantly inhibited (i) the induction of phosphorylated H2AX in S phase at 1 h post-UV (Fig. 6C) as well as (ii) 6–4PP excision during S relative to G0/G1 or G2/M (Fig. 6D). However we note that siRNA-mediated downregulation of Chk1 kinase exerted no effect on removal of this photoproduct (Fig. 6D).

## Discussion

Prior studies exploited diverse experimental systems to functionally evaluate the rational expectation that reduced NER efficiency constitutes a risk factor for melanoma development in the general population. In support of this expectation, relative to the situation for disease-free controls, primary lymphocytes derived from melanoma patients were shown to manifest defective host-cell reactivation of gene expression from UV-damaged plasmids, i.e., an indirect indicator of reduced DNA repair capacity [33], [34]. However dissenting investigations utilizing either human skin *in situ* [35] or a well-established animal melanoma model [36], in conjunction with methods that directly quantify UV photoproduct removal, indicated no relationship between NER capacity and individual melanoma susceptibility. Consistent with the latter two studies, and in apparent direct conflict with our own results herein, Gaddameedhi *et al*. showed that most human melanoma cell lines are as proficient in NER as normal primary melanocytes thereby indicating that melanoma is not frequently characterized by defects in UV damage repair [12]. However this investigation, and indeed the vast majority of previous NER studies, did not evaluate repair as a function of cell cycle. Here we utilized a flow cytometry-based assay to show that a majority of human melanoma cells lines is characterized by a significant defect in removal of UV-induced DNA photoproducts exclusively during S phase. Not surprisingly, given the obvious implications of failure to efficiently remove such photoproducts during DNA replication, SPR-deficient strains relative to SPR-proficient counterparts (i) are significantly more sensitive to the cytotoxic effects of UV and (ii) exhibit higher rates of apoptosis, as indicated by increased proportion of sub-G1 cells post-UV. Moreover, in line with our previous study in primary human lung fibroblasts, we provide evidence that defective SPR in melanoma cells correlates with reduced activation post-UV of ATR kinase, a master regulator of the cellular response to DNA damage-induced replication stress.

Since ATR is essential for cell growth, viable situations characterized by defective ATR signaling can arise solely through mechanisms that decrease rather than completely eliminate levels of active protein. This is reflected by patients with the rare genetic disorder Seckel syndrome, to date originating from a small number of families carrying germline mutations in either *ATR*, or its interacting partner *ATRIP*, that result in reduced ATR protein levels [19], [37]. Although it remains unclear whether Seckel syndrome is characterized by cancer predisposition, knockout mice modeling the human disease are not tumour prone [38], [39]. Nonetheless heterozygous ATR gene mutations have been implicated in some human cancer types that also happen to exhibit microsatellite instability [40], [41], [42], where ATR appears to constitute a haploinsufficient tumour suppressor [43]. More generally burgeoning evidence supports the idea that for certain tumour suppressors (eg., PTEN) haploinsufficiency, or partial inactivation, is sufficient to foster tumourigenesis [44], [45]. In the case of ATR it has been postulated that a threshold exists whereby relatively low expression of this kinase, while compatible with cell viability, negatively impacts cell proliferation thus actually inhibiting malignant transformation. However cells with less drastic decreases in ATR protein levels may be expected to proliferate normally but, due to reduced ATR signaling, exhibit increased genomic instability thus promoting carcinogenesis [39].

The above considerations provide a basis for understanding the potential biological importance of our findings. In general, mutations are fixed during DNA replication; therefore the efficient elimination of highly-promutagenic UV DNA photoproducts prior to S phase is critical for protection against sunlight-induced cancer. For this reason alone, our data showing that NER is significantly inhibited exclusively during S in a majority of human melanoma cell lines may harbour important implications for melanoma pathogenesis. In addition our overall data suggest that this characteristic NER defect is attributable to partial abrogation of ATR signaling, prompting speculation that ATR may constitute a haploinsufficient tumour suppressor for melanoma. In this respect, it is noteworthy that large-scale whole-exome sequencing studies have not revealed mutations within the ATR coding region in primary melanomas [46], [47]. However we show here that one among the eleven SPR-deficient melanoma strains, i.e., WM3248, display greatly reduced ATR protein levels compared with SPR proficient counterparts (Fig. 5D). This would be consistent with a hypomorphic intronic splice-site mutation in ATR, like the case for Seckel syndrome cells mentioned above. WM3248 also displayed lower levels of ATRIP in accord with studies showing co-dependence for ATR/ATRIP stability [37], [48]. This strain may therefore carry a hypomorphic mutation in ATRIP rather than ATR. In any case, a considerable number of other possible mechanisms that diminish ATR activation/signaling might be expected to engender defective SPR, possibly contributing to melanomagenesis, and these remain to be characterized in future studies. Interestingly one recent report showed that the CDC42 homologue RhoJ and its effector PAK1 modulate ATR activity via degradation of claspin after DNA damage thereby uncoupling ATR from Chk1; moreover RhoJ appears to be upregulated in advanced melanoma [49].

The precise mechanism underlying any requirement for robust ATR activity in SPR remains to be elucidated. As alluded to in the Introduction, it was recently shown that siRNA-mediated knockdown of PTEN significantly inhibits NER efficiency during all phases of the cell cycle in transformed human keratinocytes [15]. Since our three SPR-proficient melanoma strains all express functional PTEN [50], [51], [52] (Table S1), we initially speculated that downregulation of this tumour suppressor might abrogate NER in melanoma cells possibly in an S phase-specific manner. However we provide evidence that PTEN depletion in SPR-proficient WM35 and WM1366, or complete loss of PTEN in colon carcinoma cells, exerts no apparent influence on the removal of either 6–4PPs or CPDs (Fig. S3). In addition, as deduced from information displayed in Table S1, we note no obvious correlation between SPR deficiency and alterations in other genes frequently mutated in melanoma such as BRAF, N-ras, c-Kit, and CDK4.

We have also shown that depletion of Chk1 does not influence UV photoproduct removal in melanoma cells, suggesting the participation of a substrate directly modified by ATR. In this respect we note a large-scale proteomics study indicating that XPC is phosphorylated on two ATR S/T-Q consensus sites (S350 and S892) during genotoxic stress [16]. Moreover XPA was shown to directly interact with ATR, and to be phosphorylated by this kinase on S196 post-UV [53], [54]. Evidence was subsequently presented that this phosphorylation event is required for proper XPA stability following UV irradiation [55]. These latter investigations also showed that an S196A XPA mutant was unable to rescue defective NER in XPA−/− skin fibroblasts. However in contrast with the present study, DNA photoproduct removal was not evaluated specifically in S phase populations. The possibility that ATR-dependent phosphorylation of various substrates, including XPA and/or XPC, might regulate NER exclusively during S phase specifically in melanoma cells is currently under investigation.

## Supporting Information

Figure S1 **Evaluation of cell cycle progression in melanoma cell lines after UV was performed as in** **Figure 2** **, except that data for unlabeled samples (no BrdU) are not shown.**
(PDF)

Figure S2 **Pharmacological inhibition of ATR causes defective SPR in melanoma cells.** The three SPR-proficient lines in our collection were pre-treated for 1 h with 10 mM caffeine, 30 µM wortmannin (wort) or mock-treated. Under these conditions caffeine inhibits ATM and ATR, while wortmannin inhibits ATM and DNA-PK, but not ATR. Cells were refed with fresh medium containing inhibitors for post-irradiation incubations. **A**) Cell cycle-specific induction of γH2AX was measured at 30 min after 6 Gy of ionizing radiation. **B**) Same as in A, but 1 h post-UVC, as in Figure 5. **C**) Excision of 6–4PP at 6 h post-UVC, as in Figure 1. * p<0.001, two-tailed paired t-test comparing the extent of 6–4PP excision in G1 vs S.
(PDF)

Figure S3 **Influence of PTEN downregulation on NER efficiency in melanoma and colorectal carcinoma cells.** **A**) SPR-proficient WM35 cells were treated with PTEN siRNA or non-targeting siRNA control, as described in Materials and Methods. Left-panel, western blot showing the extent of PTEN knockdown and concomitant increase in p-Akt (S473). Middle panel, excision of 6–4PP at 6 h post-UVB (300 J/m^2^). Right panel, excision of CPDs at 12 h and 24 h post-UVB (200 J/m^2^). **B**) Same as A, but for SPR-proficient WM1366. **C**) Same as A but for HCT116 PTEN-null vs. isogenic PTEN wild-type control (strains kindly supplied by Dr. Todd Waldman). These cell lines were maintained in McCoy's media with 10% FBS (Lee C, Kim JS, Waldman T (2004) PTEN gene targeting reveals a radiation-induced size checkpoint in human cancer cells. Cancer Res 64: 6906-14). Antibodies used were: PTEN (sc-7974) from Santa Cruz; Akt (9272) and pAkt/Ser473 (9271) from Cell Signaling Technology and actin (ab8227-50) from Abcam.
(PDF)

Table S1 **List of melanoma cell lines used in this study.**
(PDF)
