# Supplementary figures and images for: A Majority of Human Melanoma Cell Lines Exhibits an S Phase-Specific Defect in Excision of UV-Induced DNA Photoproducts
Source: PLoS One. 2014 Jan 8;9(1):e85294. doi: 10.1371/journal.pone.0085294 (PMC3885708; doi:10.1371/journal.pone.0085294)

6h post-BrdU

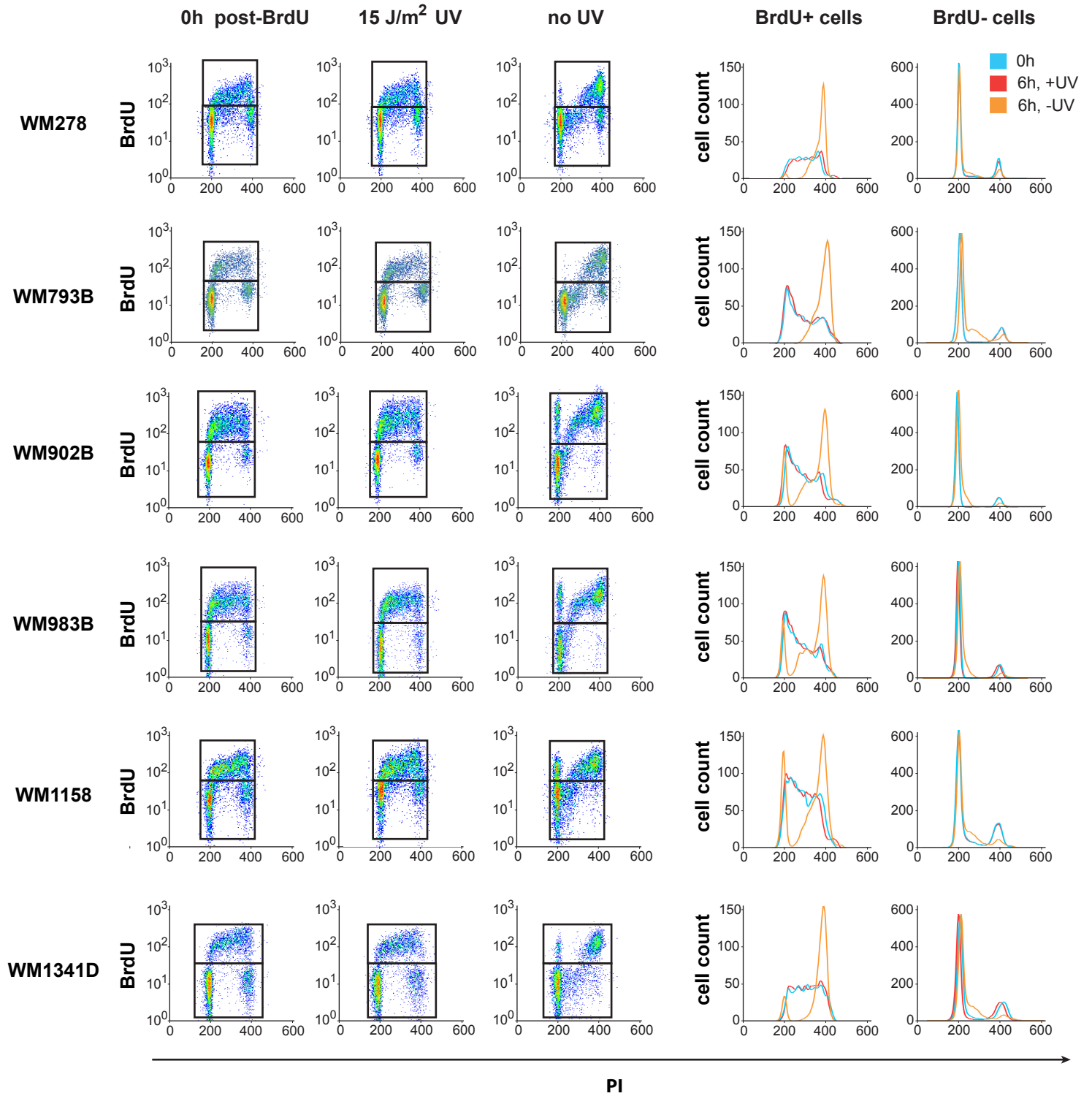

Figure S1

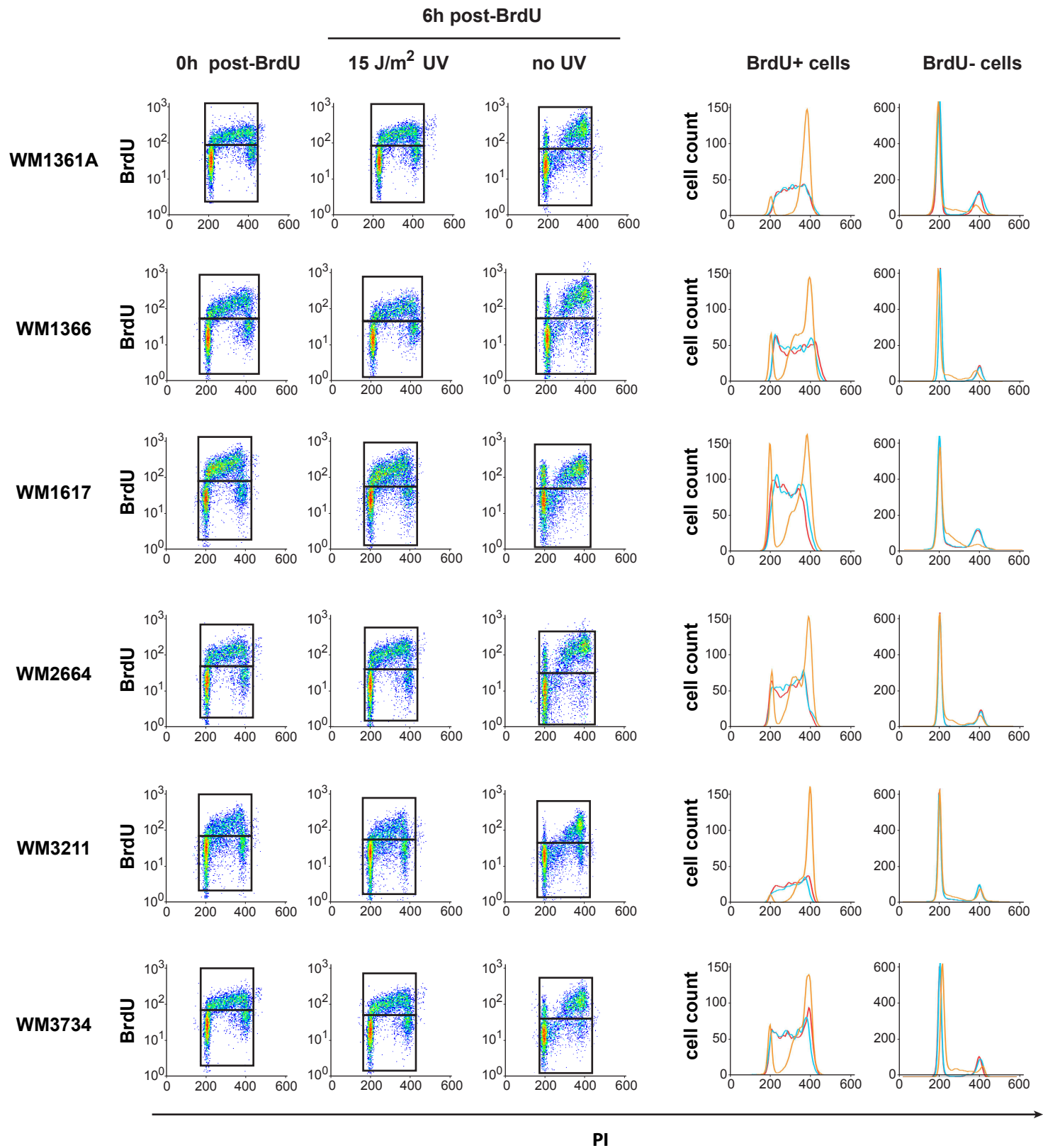

**Figure S1 (continued)**

Supplement: Figure S1 — Evaluation of cell cycle progression in melanoma cell lines after UV was performed as in Figure 2 , except that data for unlabeled samples (no BrdU) are not shown. (PDF) [file pone.0085294.s001.pdf]

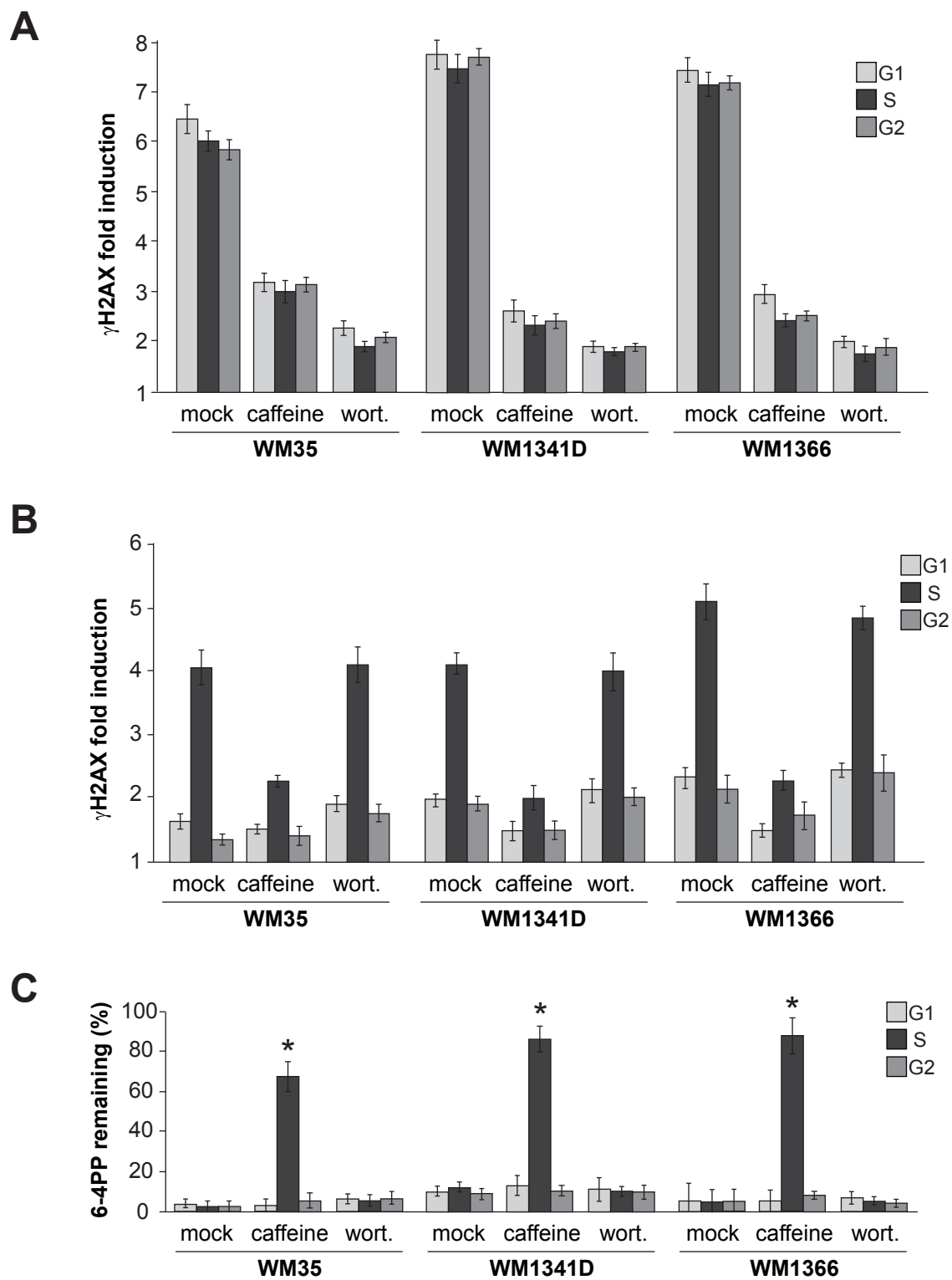

**Figure S2**

Supplement: Figure S2 — Pharmacological inhibition of ATR causes defective SPR in melanoma cells. The three SPR-proficient lines in our collection were pre-treated for 1 h with 10 mM caffeine, 30 µM wortmannin (wort) or mock-treated. Under these conditions caffeine inhibits ATM and ATR, while wortmannin inhibits ATM and DNA-PK, but not ATR. Cells were refed with fresh medium containing inhibitors for post-irradiation incubations. A) Cell cycle-specific induction of γH2AX was measured at 30 min after 6 Gy of ionizing radiation. B) Same as in A, but 1 h post-UVC, as in Figure 5. C) Excision of 6–4PP at 6 h post-UVC, as in Figure 1. * p<0.001, two-tailed paired t-test comparing the extent of 6–4PP excision in G1 vs S. (PDF) [file pone.0085294.s002.pdf]

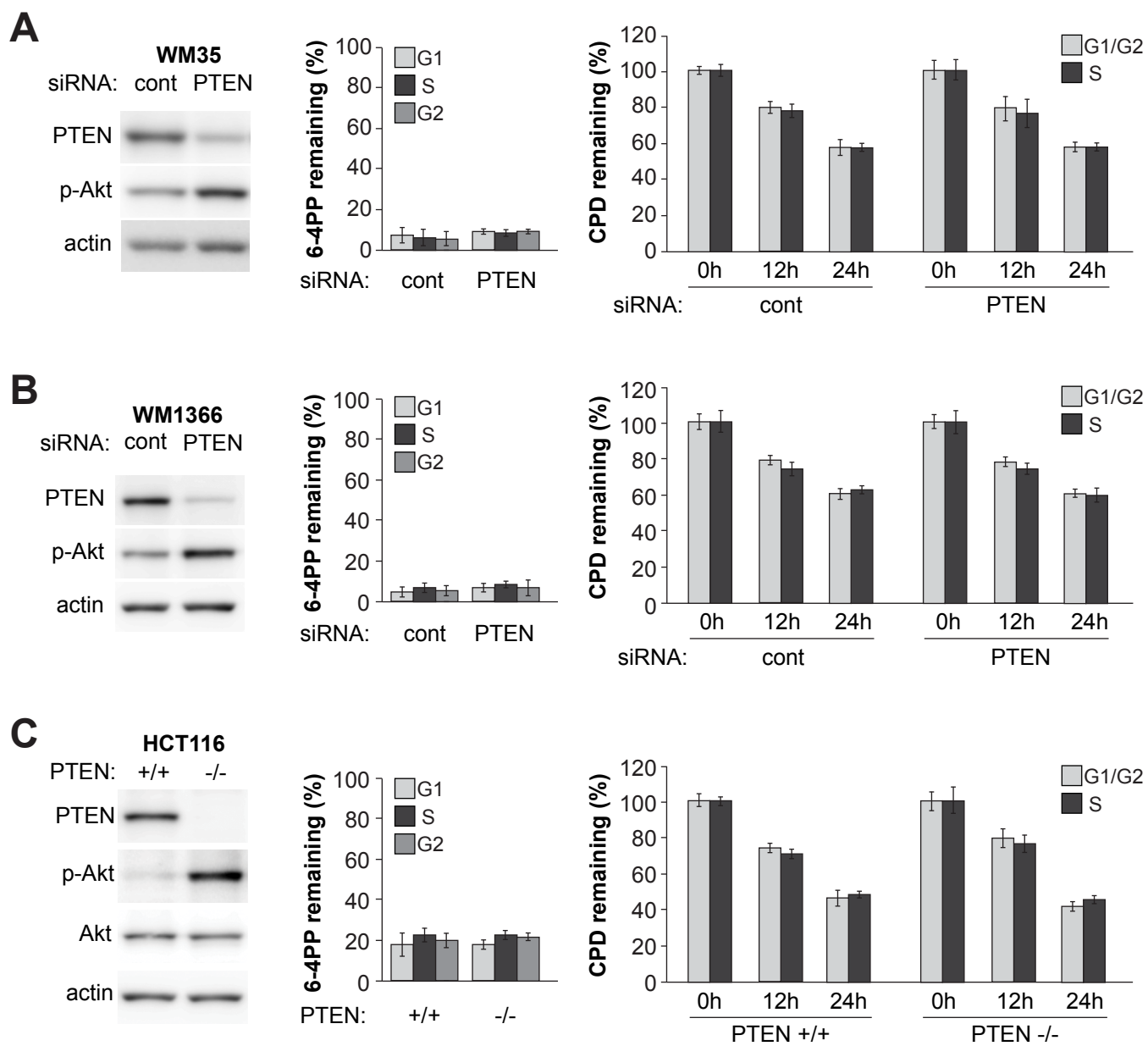

**Figure S3.**

Supplement: Figure S3 — Influence of PTEN downregulation on NER efficiency in melanoma and colorectal carcinoma cells. A) SPR-proficient WM35 cells were treated with PTEN siRNA or non-targeting siRNA control, as described in Materials and Methods. Left-panel, western blot showing the extent of PTEN knockdown and concomitant increase in p-Akt (S473). Middle panel, excision of 6–4PP at 6 h post-UVB (300 J/m2). Right panel, excision of CPDs at 12 h and 24 h post-UVB (200 J/m2). B) Same as A, but for SPR-proficient WM1366. C) Same as A but for HCT116 PTEN-null vs. isogenic PTEN wild-type control (strains kindly supplied by Dr. Todd Waldman). These cell lines were maintained in McCoy's media with 10% FBS (Lee C, Kim JS, Waldman T (2004) PTEN gene targeting reveals a radiation-induced size checkpoint in human cancer cells. Cancer Res 64: 6906-14). Antibodies used were: PTEN (sc-7974) from Santa Cruz; Akt (9272) and pAkt/Ser473 (9271) from Cell Signaling Technology and actin (ab8227-50) from Abcam. (PDF) [file pone.0085294.s003.pdf]
