# Supplementary material for: A Majority of Human Melanoma Cell Lines Exhibits an S Phase-Specific Defect in Excision of UV-Induced DNA Photoproducts
Source: PLoS One. 2014 Jan 8;9(1):e85294. doi: 10.1371/journal.pone.0085294 (PMC3885708; doi:10.1371/journal.pone.0085294)
Supplement: Table S1 — List of melanoma cell lines used in this study. (PDF) [file pone.0085294.s004.pdf]

	SPR	BRAF	PTEN gene	PTEN protein	N-ras	c-Kit	CDK4	Age	Gender	Stage	Site	Pathology	Ref.
WM35	+	V600E	Hem Del	+	WT	WT	WT	24	F	Stage 1	Scalp/neck	SSM, RGP/VGP	1, 2, 3
WM1341D	+	V600R	WT	+	WT	WT	WT	85	M	na	na	LMM, RGP/VGP	1, 2
WM1366	+	WT	WT	+	Q61L	WT	WT	79	M	na	na	VGP	1, 2
WM278	-	V600E	Hem Del	+	WT	WT	WT	62	F	Stage 2	na	NM, VGP only	1, 2, 4
WM1158	-	V600E	Hem Del	-	WT	WT	WT	32	M	Stage 2, met	Abdomen	SSM, RGP/VGP	1, 2, 5
WM2664	-	V600D	Hom Del	-	WT	WT	WT	58	F	na	Thigh skin	na	1, 2, 3
WM793B	-	V600E	Hem Del, W274X	-	WT	WT	K22Q	37	M	Stage 1	Sternum	SSM, RGP/VGP	1, 2, 3
WM983B	-	V600E	WT	+	WT	WT	WT	na	M	na	Lymph node, Met	na	1, 2
WM3248	-	V600E	K144X	-	WT	na	na	68	M	na	na	VGP	1, 2
WM3211	-	WT	na	na	WT	na	na	74	M	Stage 1	Ankle	RGP/VGP	1, 2
WM1361A	-	V600E	na	na	mut	na	na	50	M	Stage 4	Forearm	SSM, RGP/VGP	1, 2
WM902B	-	V600E	na	na	WT	na	na	na	F	na	na	SSM, VGP	1, 2
WM1617	-	V600E	na	na	na	na	na	62	F	na	Lymph node, Met	na	1, 2
WM3734	-	na	na	na	na	na	na	na	na	na	Brain	na	2

SPR-proficient and -deficient lines are highlighted in blue and yellow, respectively. Abbreviations: WT, wild-type; Hem Del, hemizygous deletion; Hom Del, homozygous deletion; M, male; F, female; SSM, superficial spreading melanoma; RGP, radial growth phase; VGP, vertical growth phase; NM, nodular melanoma; LMM, lentigo malignant melanoma; Met, metastatic; na, not available.

Supplementary references:

1. The Wistar Institute, http://www.wistar.org/lab/meenhard-herlyn-dvm-dsc/page/melanoma-cell-lines-0

2. Coriell Institute, http://ccr.coriell.org/Sections/Collections/WISTAR/CellLines.aspx?PgId=572&coll=WC

3. Paraiso KH, Xiang Y, Rebecca VW, Abel EV, Chen YA *et al.* (2011) PTEN loss confers BRAF inhibitor resistance to melanoma cells through the suppression of BIM expression. Cancer Res 71: 2750-60.

4. Spofford LS, Abel EV, Boisvert-Adamo K, Aplin AE (2006) Cyclin D3 expression in melanoma cells is regulated by adhesion-dependent phosphatidylinositol 3-kinase signaling and contributes to G1-S progression. J Biol Chem 281: 25644-51.

5. Alfano RW, Leppla SH, Liu S, Bugge TH, Herlyn M *et al.* (2008) Cytotoxicity of the matrix metalloproteinase–activated anthrax lethal toxin is dependent on gelatinase expression and B-RAF status in human melanoma cells. Mol Cancer Ther 7: 1218-26.
